# Effects of the Dopamine D2 Allosteric Modulator, PAOPA, on the Expression of GRK2, Arrestin-3, ERK1/2, and on Receptor Internalization

**DOI:** 10.1371/journal.pone.0070736

**Published:** 2013-08-06

**Authors:** Dipannita Basu, Yuxin Tian, Jayant Bhandari, Jian Ru Jiang, Patricia Hui, Rodney L. Johnson, Ram K. Mishra

**Affiliations:** 1 Department of Psychiatry and Behavioural Neurosciences, McMaster University, Hamilton, Ontario, Canada; 2 Department of Medicinal Chemistry, University of Minnesota, Minneapolis, Minnesota, United States of America; Tohoku University, Japan

## Abstract

The activity of G protein-coupled receptors (GPCRs) is intricately regulated by a range of intracellular proteins, including G protein-coupled kinases (GRKs) and arrestins. Understanding the effects of ligands on these signaling pathways could provide insights into disease pathophysiologies and treatment. The dopamine D2 receptor is a GPCR strongly implicated in the pathophysiology of a range of neurological and neuropsychiatric disorders, particularly schizophrenia. Previous studies from our lab have shown the preclinical efficacy of a novel allosteric drug, 3(R)- [(2(S)-pyrrolidinylcarbonyl)amino]-2-oxo-1-pyrrolidineacetamide (PAOPA), in attenuating schizophrenia-like behavioural abnormalities in rodent models of the disease. As an allosteric modulator, PAOPA binds to a site on the D2 receptor, which is distinct from the endogenous ligand-binding site, in order to modulate the binding of the D2 receptor ligand, dopamine. The exact signaling pathways affected by this allosteric modulator are currently unknown. The objectives of this study were to decipher the *in vivo* effects, in rats, of chronic PAOPA administration on D2 receptor regulatory and downstream molecules, including GRK2, arrestin-3 and extracellular receptor kinase (ERK) 1/2. Additionally, an *in vitro* cellular model was also used to study PAOPA’s effects on D2 receptor internalization. Results from western immunoblots showed that chronic PAOPA treatment increased the striatal expression of GRK2 by 41%, arrestin-3 by 34%, phospho-ERK1 by 51% and phospho-ERK2 by 36%. Results also showed that the addition of PAOPA to agonist treatment in cells increased D2 receptor internalization by 33%. This study provides the foundational evidence of putative signaling pathways, and changes in receptor localization, affected by treatment with PAOPA. It improves our understanding on the diverse mechanisms of action of allosteric modulators, while advancing PAOPA’s development into a novel drug for the improved treatment of schizophrenia.

## Introduction

Modulation of G protein-coupled receptor (GPCR) signaling represents an important mechanism of action for a range of therapeutics. Consequently, recent scientific studies have focussed on modulating GPCR activity with the use of a novel range of allosteric modulators. These ligands bind to a site distinct from that of the endogenous ligand, thereby activating novel pathways to modulate receptor signaling. Allosteric sites display a much higher sequence divergence compared to orthosteric sites, allowing for the design of highly receptor-specific ligands. Additionally, they provide a unique method to subtly modify receptor activity to varying degrees, allowing for a more fine-tuned control of signaling events [1–5].

**Figure 1:**
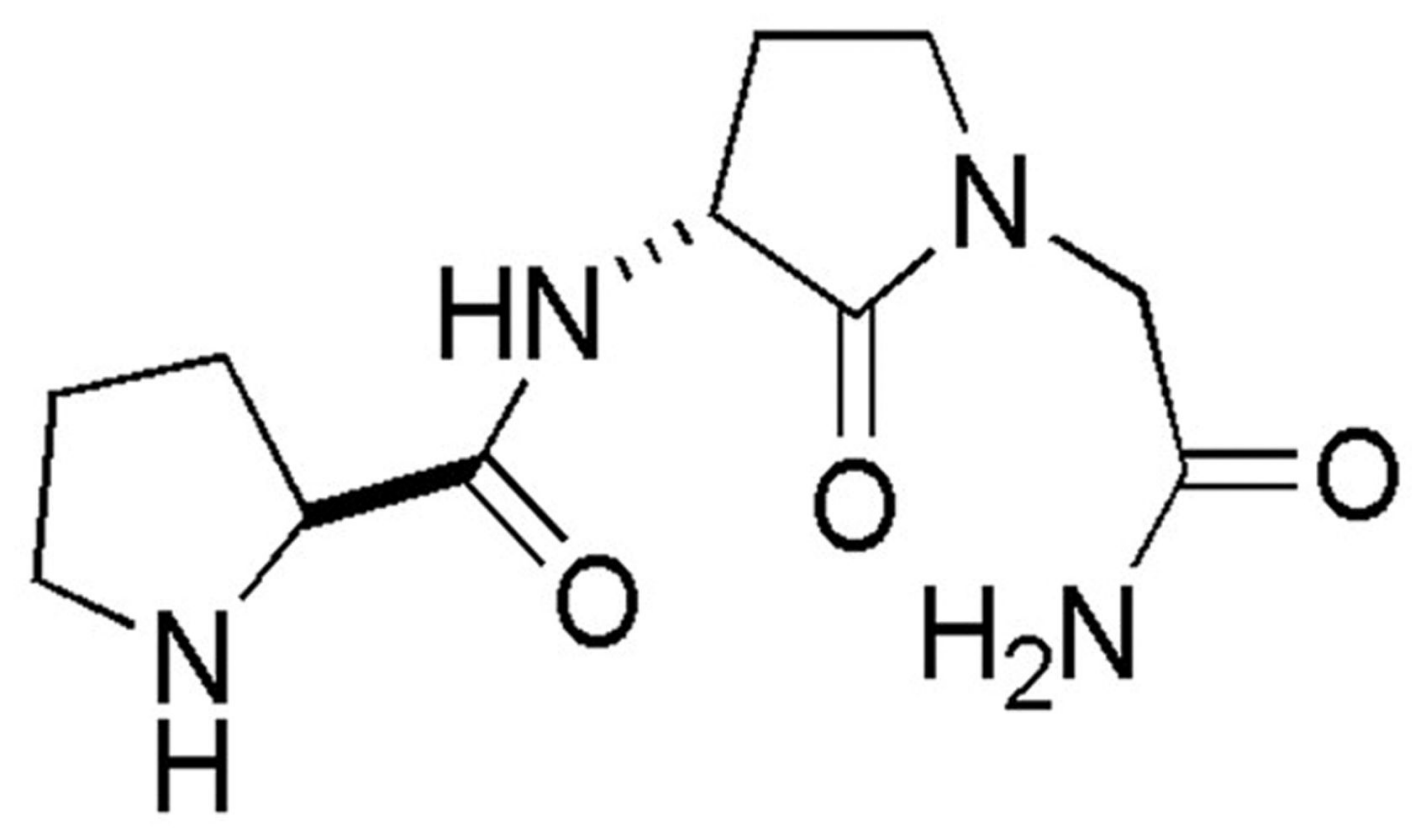
Chemical structure of the dopamine D2 receptor allosteric modulator 3(R)-[(2(S)-pyrrolidinylcarbonyl) amino]-2-oxo-1-pyrrolidineacetamide (PAOPA).

Previous studies from our lab have developed novel allosteric ligands targeting the dopamine D2 receptor, based on the endogenous tripeptide, L-prolyl-L-leucyl-glycinamide (PLG) [6–19]. The D2 GPCR is expressed in various neuronal regions, including the striatum, nucleus accumbens and substantia nigra, and is highly implicated in human psychiatric and neurological disorders, including schizophrenia and Parkinson’s disease [20–23]. The allosteric ligand demonstrating the highest preclinical efficacy in our studies is the conformationally constrained PLG peptidomimetic, 3(R)- [(2(S)-pyrrolidinylcarbonyl) amino]-2-oxo-1-pyrrolidineacetamide (PAOPA) (Figure **1**) [24,25]. At the preclinical level, PAOPA has been shown to be effective in attenuating behavioural abnormalities in rodent models of schizophrenia, including the amphetamine-sensitized and dizocilpine (MK-801)-sensitized disease models [26,27]. Previous studies have shown PAOPA to interact with a unique allosteric site on the dopamine D2 receptor, to facilitate agonist binding by increasing the proportion of D2 receptors in the high affinity state [24,28]. However, the precise mechanism of action for this drug is currently unknown.

GPCR trafficking and signaling is controlled by a number of key regulatory proteins, including G protein-coupled receptor kinases (GRK), arrestins, dynamin and clathrin. Receptor overstimulation by exposure to agonists leads to receptor phosphorylation by GRKs, followed by recruitment of arrestins, and could lead to downregulation in activity by receptor internalization. Previous studies have demonstrated the dominant-negative mutations of GRK2 to abolish any D2 receptor internalization, evincing the importance of this protein in the downregulation of this receptor [29]. Moreover, arrestin-3 appears to be the isoform which when knocked out in mice models, has shown to inhibit the internalization of D2 receptors in striatal cell cultures [30]. Internalized receptors can be recycled back to the plasma membrane, or targeted for lysosomal degradation. This process plays a key role in regulating the activity level, and downstream effects of crucial GPCRs.

Pathophysiological states, as well as chronic drug treatments, can alter the expression of regulatory GRK and arrestin proteins, which can significantly affect GPCR signaling and the resulting downstream effects [31–34]. Measurement of post-mortem cortical levels has shown decreased mRNA of GRKs and arrestins, and decreased GRK3 protein levels in patients suffering from schizophrenia [35]. Chronic treatment of schizophrenia with the widely prescribed antipsychotic drugs haloperidol and clozapine, have also shown to have significant, regional-specific effects on GRK and arrestin expression [34]. These modulations can affect receptor trafficking and density, thereby having a significant effect on the downstream signaling pathways.

The objectives of this study were to examine changes in protein expression of D2 receptor-related regulatory proteins GRK2 and arrestin3 in the dopaminergic-rich region of the striatum, following chronic PAOPA treatment in rodents. To detect changes in downstream signaling associated with altered expression of the above regulatory proteins, this study also examined changes in phosphorylation levels of the effector molecule belonging to the mitogen-activated protein kinase (MAPK) family, specifically the extracellular signal regulated kinase (ERK) 1/2 molecule. Finally, this study also examined the effects of these expression changes on D2 receptor internalization in a cellular model. This study was able to demonstrate that chronic PAOPA treatment caused an *in vivo* increase in the striatal expression of GRK2, arrestin-3 and phospho-ERK1/ERK2, while also enhancing D2 receptor internalization in *in vitro* models. Understanding the chronic effects of PAOPA on these signaling proteins and on D2 receptor trafficking can provide a better understanding of its mechanism of action, as well as improve our knowledge on the etiology and future treatment of schizophrenia.

## Materials and Methods

### Ethics statement

All studies were approved by the McMaster Animal Research Ethics Board and were in compliance with the guidelines of the Canadian Council on Animal Care (Animal utilization protocol # 10-08-59). All cell culture work was approved by the Presidential Biosafety Advisory Committee (Permit # 2011-079).

### Animals, drug regimen and tissue collection

Twelve adult male Sprague Dawley rats (250 g) were obtained from Charles River Laboratories (Wilmington, MA) and individually housed at the McMaster Central Animal Facility. Animals were maintained under constant temperature and humidity, with a reverse 12:12 light/ dark cycle, and *ad libitum* access to food and water. The dopamine D2 allosteric peptide PAOPA (US patent: PCT/CA2011/000968) was synthesized in the laboratory of Dr. Rodney L. Johnson (University of Minnesota, MN) as described previously [25]. Fresh peptide solutions were prepared daily at a 1 mg/mL concentration. Rats (n= 6/group) were treated daily, for 45 days, with a chronic administration of PAOPA via intraperitoneal (I.P.) route at a dose of 1 mg/kg. One hour following the final drug administration, rats were anaesthetized with isofluorane, and sacrificed by decapitation. Rat brains were removed, the striatum and cerebellum dissected over ice, and stored at -80^°^ C until use.

### Western Immunoblotting

Collected rat striata and cerebellum were homogenized using a pestle in phosphate-buffered saline (PBS) with Complete Mini, EDTA-free protease inhibitor tablet (Hoffmann La-Roche, Mississauga, ON) and PhosStop phosphatase inhibitor cocktail tablet (Hoffmann La-Roche, Mississauga, ON). Resulting homogenate was sonicated on ice three times, for 10 seconds each, and a Bradford protein assay was used to quantify the sample protein concentration. 15 µg of sample was resuspended in 2X sodium dodecyl sulfate (SDS) sample buffer, and separated by electrophoresis on 10% or 12% acrylamide gels, using protocols as previously described by our lab [36]. Briefly, separated proteins were transferred to a 0.45 µM polyvinylidene (PVDF) membrane, blocked for 1 hour in 5% skim milk, and exposed to primary antibody overnight. Primary antibody-bound membranes were exposed to secondary antibody for 1.5 hours at room temperature, and visualized using enhanced chemiluminescence (ECL) substrates. GRK2 was detected using monoclonal anti-GRK2 antibody produced in rabbit (1:2000; Abcam, Cambridge, MA), and arrestin-3 was detected using monoclonal anti-ARRB2 antibody produced in mouse (1:1000; Sigma, St. Louis, MO). Phosphorylation of downstream molecule ERK1/2 was measured using polyclonal phospho-specific anti- p44/42 MAPK (ERK1/2) (Thr202/Tyr404) produced in rabbit (1:2000; Cell signaling, Boston, MA). Glyceraldehyde-3-phosphate dehydrogenase (GAPDH) was used as a housekeeping gene to normalize changes in protein expression and was detected using anti-GAPDH antibody produced in mouse (1:10000; Millipore, Billerica, MA). The anti-rabbit exposed membranes were incubated with anti-rabbit IgG horseradish peroxidase (HRP)- linked whole secondary antibody from donkey (1:5000, Sigma Aldrich, St. Louis, MO), and anti-mouse exposed membranes were incubated with anti-mouse IgG HRP-linked whole secondary antibody from sheep (1:5000; Sigma Aldrich, St. Louis, MO).

### Live cell microscope imaging

A cellular model was kindly provided by Dr. J. Javitch (Columbia University, New York) to study D2 receptor internalization, with details provided in a previous paper [37]. Briefly, this model was a tetracycline-regulated expression cellular line (T-REx-293) (Invitrogen, Carlsbad, CA), expressing bovine GRK2 in pcDNA4/TO vector, rat arrestin-3 in pcDNA5/TO vector, and human D2 receptor with a FLAG epitope and fused with enhanced yellow fluorescent protein (eYFP) in pCIN4 vector (Invitrogen, Carlsbad, CA). The co-expression of GRK2 and arrestin-3 was to allow for the availability of adequate machinery in order to internalize the overexpressed D2/eYFP. Cells were grown at 37^°^ C with 5% CO_2_ in Dulbecco’s modified Eagle’s medium (DMEM) containing 10% fetal calf serum and 5 mg/mL blasticidin. The positive stable expression in the T-REx-293 cellular system of all three proteins, including GRK2, arrestin-3 and D2/eYFP, was confirmed by immunoblotting in our lab (data not shown). To visualize D2 receptor internalization using live cell imaging, cells were seeded on 25 mm poly-lysine coated coverslips, and allowed to grow for 48 hours (Neuvitro, El Monte, CA). 24 hours prior to microscope visualization, cells were treated with 1 µg/mL tetracycline to induce the overexpression of GRK2 and arrestin-3. Images were acquired by a 63X glycerol immersion objective using a confocal microscope Leica DMI 6000B (Wetzlar, Germany), equipped with a Hamamatsu C9100-12 back-thinned EMCCD camera. To maintain cell viability, images were captured in a Neue LiveCell2 whole microscope chamber, which maintained a well-regulated environmental temperature of 37^°^ C and CO_2_ of 5%. Drug treatments included 30 µM quinpirole (Sigma, St. Louis, MO), and 10 µM PAOPA. These were diluted in DMEM and perfused immediately at the start of visualization onto the cells, by mounting the coverslip with the cells in an Attoflour cell chamber (Invitrogen, Carslbad, CA). Cell images were acquired every 2 minutes for 30 minutes using the Volocity 4 acquisition software.

### [^3^H]-sulpiride binding assay

[^3^H]-sulpiride assays have previously been conducted by other labs as a method to quantify D2 receptor internalization in cellular models [29,38–40]. In this study, these assays were conducted by seeding the triple transfected T-REx-293 cells expressing GRK2, arrestin-3 and D2/eYFP, onto poly-lysine coated 24-well plates at a density of 2X10^5^ cells/well, and allowing them to grow for 48 hours. 24 hours prior to the assay, cells were induced to overexpress GRK2 and arrestin-3 by adding a final concentration of 1 µg/mL tetracycline. Cells were treated for 1.5 hours in DMEM media, with 20 mM 4-(2-hydroxyethyl)-1-piperazineethanesulfonic acid (HEPES) and 0.2 mM sodium metabisulfite. Treatments included combinations of 30 µM quinpirole (Sigma, St. Louis, MO), and/or PAOPA, as written in the results section. Drug treatments were ceased by rinsing the cells gently three times with ice-cold Earle’s buffered saline solution (EBSS). Cells were then bound to 6 nM of [^3^H]-sulpiride (PerkinElmer Life, Waltham, MA) diluted in EBSS, for 3.5 hours at 4^°^ C. [^3^H]-sulpiride is a D2 receptor-like selective antagonist, with maximal inhibition observed at D2 and D3 receptors [41,42]. Additionally, as a highly hydrophilic compound, sulpiride only interacts and tags membrane receptors, disregarding any cytoplasmic internalized receptor, thereby allowing for a quantification of receptor internalization. Following radioligand binding, cells were once again rinsed three times with ice-cold EBSS, to remove any unbound radioactivity. Bound, intact cells were lifted using 1% Triton-X, placed into a scintillation vial with cocktail and counted for radioactivity using a scintillation counter Model LS5KTA (Beckman Coulter, Mississauga, ON). Percent D2 receptor internalization was calculated using the decays per minute (DPM) count, comparing control cells to the treated cells.

### Data Analysis

Intensity of protein bands was quantified using ImageJ 1.43M (National Institutes of Health). Student’s t-test was used to compare differences in protein expression between control and treated groups for immunoblot assays. One-way analysis of variance (ANOVA) was used in combination with Tukey’s post-hoc analysis to compare differences between treatment groups for the [^3^H]-sulpiride binding assays. An expression change was considered significant at p < 0.05. Data are presented as the percent change compared to control ± SEM.

## Results

### Effect of PAOPA on the expression of GRK2 and arrestin-3 in the striatum

**Figure 2:**
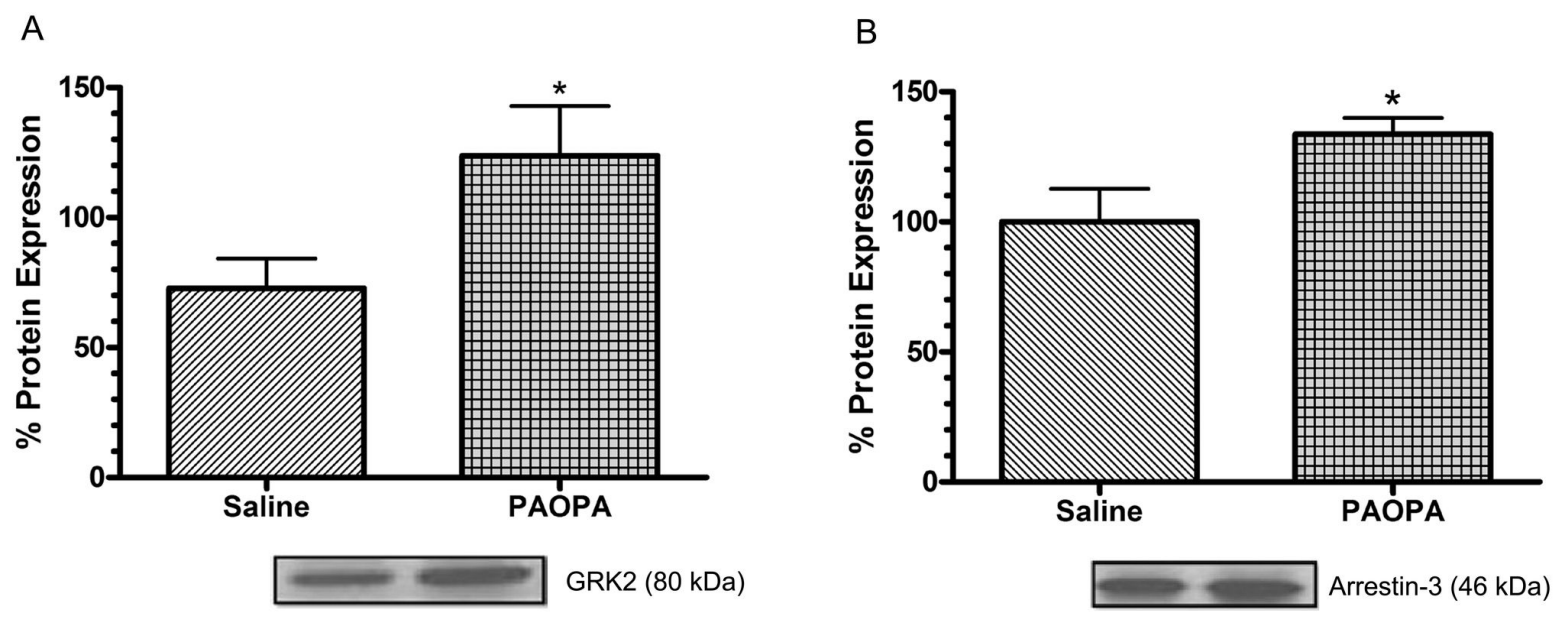
Effect of chronic PAOPA administration on expression of GPCR regulatory proteins in rat striatum. PAOPA (1mg/kg) was chronically administered to rats, and immunoblotting was used to quantify changes in expression of proteins involved in the downregulation process of the dopamine D2 receptor. Chronic PAOPA treatment increased the expression within the striatum of (A) G protein-coupled receptor kinase 2 (GRK2) by 41% and of (B) arrestin-3 by 34%. Data are shown with representative immunoblots, and expressed as a percentage of control ± SEM where *p < 0.05 (Student’s t-test).

GPCR signaling is regulated by key molecules, including GRKs and arrestins, the expressions of which can be affected by long-term ligand modulation [31–34]. To determine whether protracted treatment with PAOPA results in changes in the expression of these molecules, rats were chronically treated with this D2 allosteric modulator. Chronic treatment of rodent models with the conformationally-constrained PLG peptidomimetic, PAOPA, caused a significant increase of GRK2 expression in the striatum, by 41%, and arrestin-3, by 34% (Figure **2**). There was no change in the expression of the housekeeping protein GAPDH, which was used to normalize the results (data not shown).

### Effect of PAOPA on the expression of ERK1/2 in the striatum

**Figure 3:**
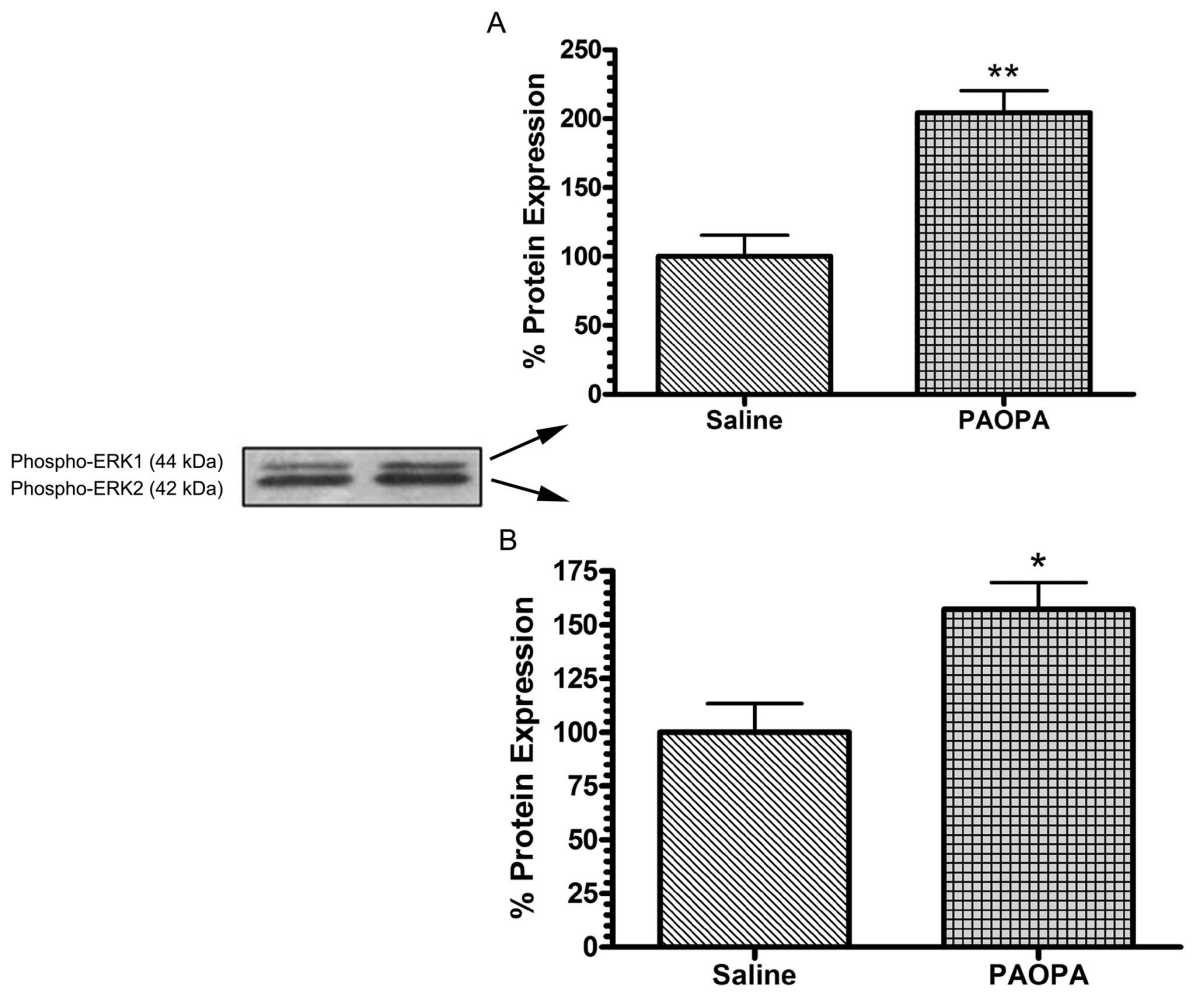
Effect of chronic PAOPA administration on expression of downstream molecules in rat striatum. PAOPA (1mg/kg) was chronically administered to rats, and immunoblotting was used to quantify changes in expression of dopamine D2 receptor-related downstream signaling proteins extracellular receptor kinase 1/2 (ERK1/2). Chronic PAOPA treatment increased the expression within the striatum of (A) ERK1 by 51% and of (B) ERK2 by 36%. Data are presented with representative immunoblots, and as a percentage of control ± SEM where *p < 0.05 and **p<0.01 (Student’s t-test).

Ligands affecting GRK and arrestin protein expression have also been shown to have effects on the mitogen-activated protein kinase (MAPK) signaling pathway [43,44]. The activation of members of the MAPK pathways, including ERK1/2, is dependent on the phosphorylation of these proteins [45]. Therefore, only the activated levels of these proteins were measured in this study, using phospho-specific antibodies for the Thr202 and Tyr404 sites for ERK1 and ERK2 respectively. In this study, PAOPA was observed to have significant effects in activating ERK function within the striatum of treated animals, leading to an increase in levels of phospho-ERK1 by 51% and phospho-ERK2 by 36% (Figure **3**). Expression of the housekeeping GAPDH protein was again analyzed within the same samples to normalize the expression of ERK1/2, and was shown to not change with PAOPA treatment (data not shown).

### Effect of PAOPA on the expression of GRK2, arrestin-3 and ERK1/2 in the cerebellum

**Figure 4:**
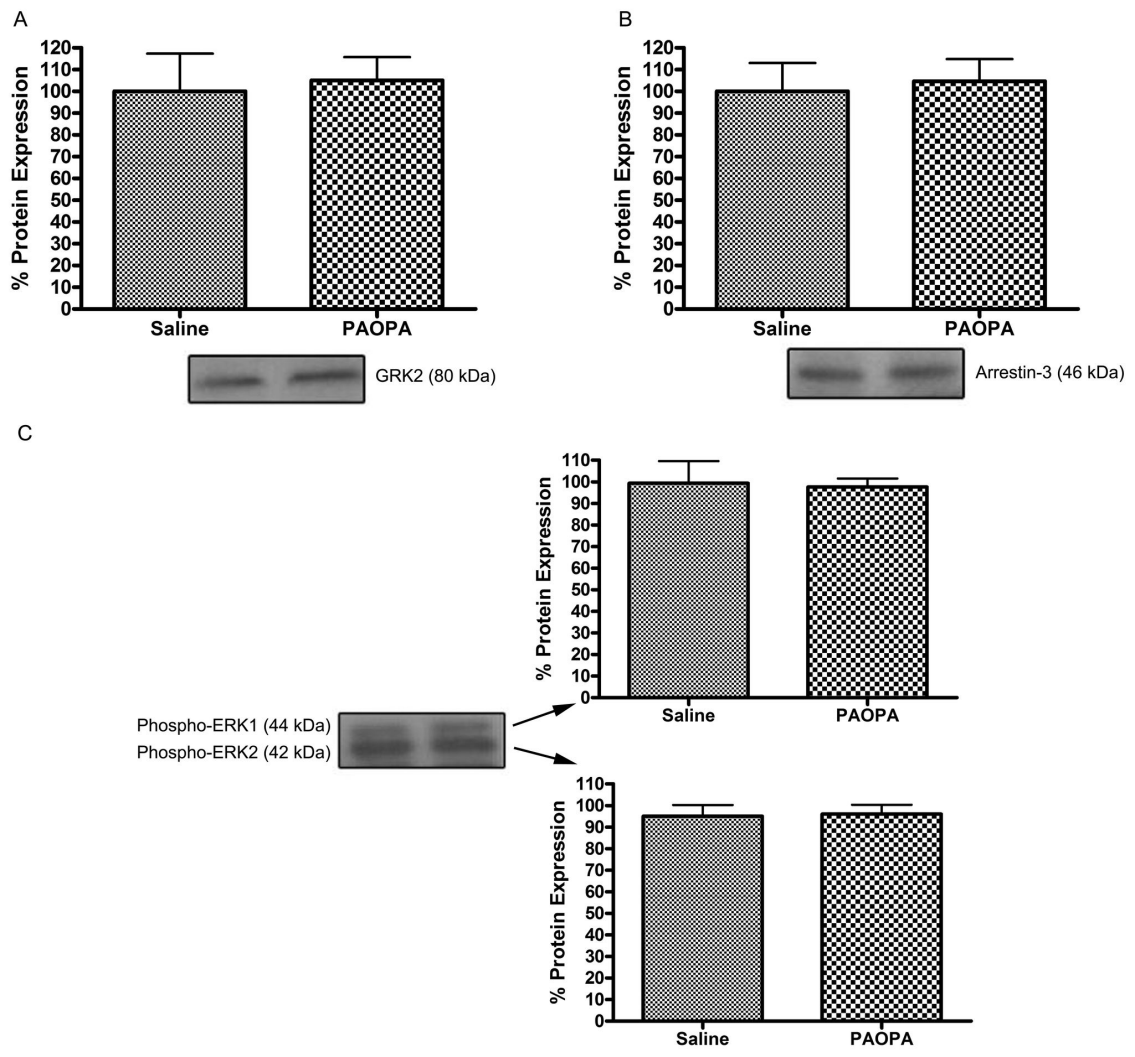
Effect of chronic PAOPA administration on expression of GPCR regulatory and downstream molecules in rat cerebellum. PAOPA (1 mg/kg) was chronically administered to rats, and immunoblotting was used to quantify changes in expression of dopamine D2 receptor-related signaling proteins G protein-coupled receptor kinase 2 (GRK2), arrestin-3 and extracellular receptor kinase 1/2 (ERK1/2). PAOPA treatment caused no significant differences in the expression within the cerebellum of (A) GRK2, (B) arrestin-3 and (C) ERK1/2. Data are presented with representative immunoblots, and as a percentage of control ± SEM.

Studies have often used the cerebellum as a region of reference to study D2 receptor-related effects, as this region is considered to have a negligible density of this receptor [46–48]. Thus, in order to relate the observed changes in protein expression in the striatum caused by PAOPA to modulation of the dopaminergic system, changes in protein expression of GRK2, arrestin-3 and phospho-ERK1/2 were measured in the cerebellum. Results, as shown in Figure **4**, demonstrate that no significant alteration in the expression of these proteins was observed in the cerebellum of chronically treated rat models. These expression levels were also normalized to expression of the housekeeping protein GAPDH, which did not show any significant changes with PAOPA treatment (data not shown).

### Effect of PAOPA on D2 receptor internalization in a cellular model

**Figure 5:**
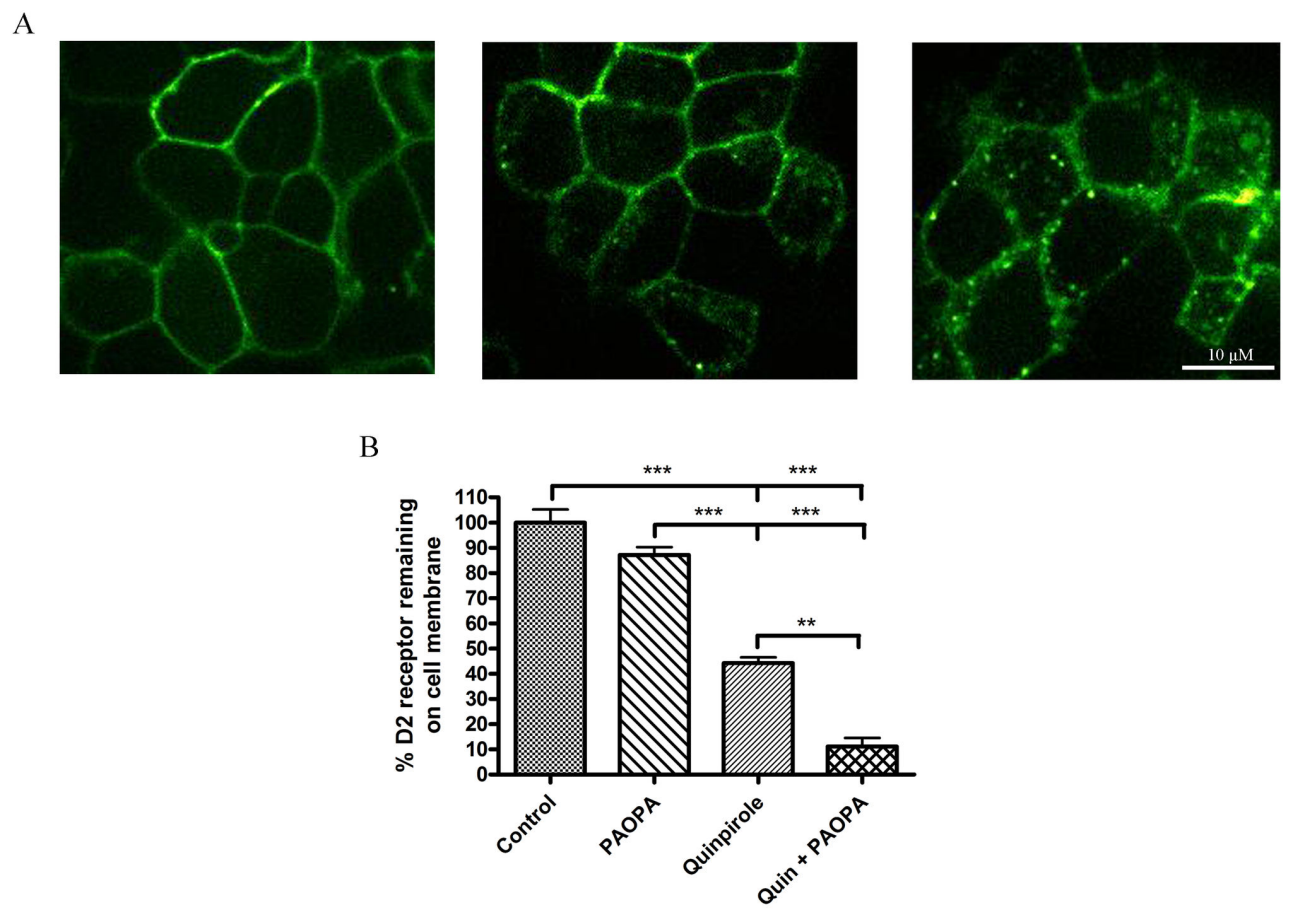
Effect of PAOPA on dopamine D2 receptor internalization in a cellular model. TREx-293 cells were stably transfected with D2/enhanced yellow fluorescence protein (eYFP), G protein-coupled receptor kinase 2 (GRK2) and arrestin-3. (A) Live cell confocal microscopy imaging of this cellular model treated as control (left panel), D2 agonist quinpirole (30 µM) (middle panel), and quinpirole with PAOPA (10 µM) (right panel) are shown. The green fluorescence represents the dopamine D2 receptor, which appears to be primarily present on the cell membrane in the control cells, and locating to intracellular regions with quinpirole, and quinpirole with PAOPA treatment. (B) [^3^H]-sulpiride binding assays were used to quantify D2 receptor internalization, and this graph shows the percent of D2 receptors remaining on the cell membranes following given treatments of quinpirole (30 µM), PAOPA (10 µM) and quinpirole with PAOPA. Results show the addition of PAOPA to increase dopamine D2 receptor internalization by ~33%. Data are presented as a percentage of control ± SEM where *** p < 0.001, ** p < 0.01 (one-way analysis of variance).

GPCR stimulation by a ligand can often result in changes in receptor localization, leading to the movement of receptors from the cell membrane to internal cellular regions, causing a downregulation of overall activity. PAOPA acts as a positive allosteric modulator of the dopaminergic system, potentiating agonist binding to the D2 receptor, and therefore, could have an effect on the internalization of this receptor [24]. Cells triply transfected with GRK2, arrestin-3 and D2/eYFP, were treated with DMEM (control), PAOPA, a D2 agonist (quinpirole) or a combination of both PAOPA and quinpirole. Confocal microscopy results in Figure **5A**, show the control cells (left panel) to express the D2/eYFP primarily on the cell membrane. Treatment with quinpirole (middle panel) illustrates D2/eYFP receptor internalization, with a certain amount of the green fluorescence appearing within the cytoplasmic region of the cell. Finally, the addition of PAOPA to the quinpirole treatment (right panel) shows increased levels of punctate D2/eYFP receptors located in the intracellular region, suggesting a possible enhancement in receptor internalization with the addition of the allosteric modulator, PAOPA. We found it challenging to quantify the level of D2/eYFP internalization, as no reliable software could be attained to automate the fluorescence counting separately for the membrane compared to the cytoplasmic region. Therefore, to supplement the visualization of D2 receptor internalization, quantification of D2 receptor internalization with PAOPA treatment was conducted using [^3^H]-sulpiride assays. Results, displayed in Figure **5B**, show that 1.5 hours following treatment, quinpirole causes ~56% internalization. The addition of PAOPA to quinpirole treatment increased internalization of the D2 receptor by ~33%, leading to ~89% internalization. PAOPA administration by itself had no significant effect on internalization, as is expected, since an allosteric modulator is defined as being quiescent in the absence of its endogenous ligand. Overall, the addition of PAOPA to an agonist treatment potentiated the ability of this agonist to cause D2 receptor internalization.

## Discussion

This study was able to demonstrate that the chronic administration of PAOPA increases protein expression of GRK2, arrestin-3, and activates, by phosphorylation, the downstream molecules ERK1 and ERK2, within the striatum. Enhanced agonist stimulation of GPCRs, as occurs with chronic PAOPA administration, have previously shown to have unique effects on the expression of the cell’s regulatory machinery of GRKs and arrestins, as well on downstream kinase molecules [49]. For example, chronic infusion with β-adrenergic agonist or antagonist leads to increases and decreases in levels of GRK2 mRNA and protein expression respectively [49]. Furthermore, previous studies have clearly demonstrated that agonist-induced activation of D2 receptors increases ERK1/2 phosphorylation, including within the striatum [50–52]. The observed increases in protein expression in our studies ranged from 34–51%. This level of change in GPCR regulatory proteins, though modest, can have functionally important effects on GPCR signaling, which can manifest as physiologically significant responses [53]. Studies in cellular systems have shown overexpression of either GRK2 or arrestin-3 to enhance agonist-induced phosphorylation and sequestration of the D2 receptor, which is further enhanced by co-expression of both GRK2 and arrestin-3 [54]. Additionally, hemizygous knockouts expressing 50% less GRK proteins in mice models leads to phenotypes similar to complete knockouts [53]. Collectively, these studies show that chronic drug treatments can have an impact on the expression of GPCR trafficking and downstream molecules, which can result in significant phenotypic changes.

The current understanding of the GPCR trafficking pathway posits that chronic activation by ligand binding leads to phosphorylation of the receptor by a GRK, allowing for arrestin binding and attenuation of further G protein signaling [53]. Activation of the GRK/arrestin system can have dual effects on physiological systems. This system can suppress G protein signaling by promoting receptor desensitization, but can also enhance non-G protein signaling via the arrestin-3-dependent pathways [43,53]. Therefore, drugs which alter the expression of these regulatory proteins cannot only suppress D2 receptor activity, but also activate novel potential pathways to modulate dopaminergic neurotransmission.

The changes observed in GRK2, arrestin-3 and ERK1/2 expression, elicited by chronic PAOPA administration, can involve quite complex mechanisms, engaging multiple pathways. In our studies, the exact pathway linking D2 receptor activation by PAOPA to the increases in ERK1/2 is yet to be determined. One suggested mechanism could involve the increased recruitment of arrestin-3 to activated receptors, leading to the formation of signaling scaffolds. These scaffolds bring in closer proximity Raf isoforms, which phosphorylate MEK within the scaffold, which in turn phosphorylates and activates ERK1/2 [43]. Increased ERK1/2 levels can have a range of cellular effects. One particular study demonstrated the involvement of the ERK1/2 pathway in the upregulation of GRKs, via activation of Sp-1 and Ap-2 transcription factors in neuronal cells [55]. Increased arrestin-3 levels could also promote formation of another key scaffold complex, involving dephosphorylation and activation of Akt using PP2A. This pathway leads to activation of glycogen synthase kinase-3 (GSK-3) which has shown to be significantly involved in neuropsychiatric disorders, including schizophrenia [56]. The observations of this study have yielded a few possible mechanisms of action of PAOPA, and opened avenues to improve the understanding of this allosteric modulator’s actions in altering dopaminergic neurotransmission.

The effects of PAOPA observed in this study can almost certainly be associated to be occurring via allosteric effects on the D2 dopaminergic system. Previous *in vitro* and *in vivo* studies have shown the specificity of PAOPA for the D2 receptor, with non-significant effect on agonist binding on the D1 and D3 receptors, as well as the α2-adrenergic receptor [24]. Radioligand binding studies have shown that PAOPA modulates agonist binding to D2 without competing with dopamine binding, suggesting an allosteric interaction [24,25]. PAOPA also has no statistically significant effect on specific binding of D2 antagonists such as [^3^H]-spiperone [24]. Additionally, in animal models (e.g., 6-hydropdopamine (6-OHDA); vacuous chewing movement models) PAOPA has shown to potentiate the activity of D2 agonists, such as apomorphine [57,58], and attenuate the effects of D2-specific antagonist haloperidol [58]. Finally, the increases in the expression of GRK2, arrestin-3 and ERK1/2 observed in this study were seen to occur within the striatum of PAOPA-treated rats. No such significant changes were observed to occur in the cerebellum of the same treated rats, which is generally used as a negative control to study D2 receptor-related effects, as it is considered to be devoid of these receptors [46–48]. Together, this implies that the observed changes in striatal protein expression can almost certainly be attributed to PAOPA’s modulatory effect on the D2 dopaminergic system.

The effect of PAOPA on the internalization of the D2 receptor, as demonstrated in the triple transfected cellular model, adds further insight into the mechanistic actions of this allosteric drug. Agonist stimulation of the D2 receptor by quinpirole caused internalization of the D2 receptor, which was further enhanced by addition of PAOPA to the agonist treatment, as shown both, by live cell imaging, and [^3^H]-sulpiride assays. As a positive allosteric modulator, PAOPA increases agonist binding to the D2 receptor, by converting low affinity (GTP bound) receptors to high affinity (GDP bound) receptors, and therefore, is expected to induce increased D2 internalization, as observed in our results [24]. Furthermore, D2 receptor internalization can help explain the biphasic dose–response curve characteristic of PAOPA, whereby, this drug increases agonist binding at lower concentrations, and decreases it at higher concentrations [24]. The decrease in agonist binding at higher concentrations of PAOPA could be related to down-regulation in the overall availability of the D2 receptor due to internalization, providing a possible explanation for the therapeutic effect of PAOPA in the hyperdopaminergic state observed consistently in schizophrenia [23].

This study focuses on the GRK-mediated internalization of the D2 receptor following treatment with PAOPA, which is internalization via the homologous desensitization pathway. However, it is worthy to note that additional pathways exist for receptor downregulation, including heterologous desensitization. This process involves the broad desensitization of receptors in a cell, following prolonged activation of other GPCRs within that cell, and is mediated by protein kinase C (PKC) for the D2 receptor [59,60]. Heterologous desensitization results in non-specific downregulation of GPCRs, whereas GRK-mediated desensitization is specific for the D2 receptor, leading this study to primarily focus on the latter [40,61]. However, subsequent studies in the future will also study heterologous desensitization in response to PAOPA treatment.

PAOPA has previously shown preclinical efficacy in the prevention and attenuation of schizophrenia-like behavioural abnormalities in rodent models of the disease [26,27]. As a potential new antipsychotic drug, the alterations caused by PAOPA in signaling pathways can be compared to that of drugs currently available in the market. For example, chronic treatment with atypical antipsychotic drug, olanzapine, enhances ERK activation in the prefrontal cortex, similar to our observations with PAOPA treatment in the striatum. Additionally, recent studies have shown a range of effects of atypical antipsychotic clozapine in a multitude of brain regions, including increases in GRK5, arrestin-2 and long-term activation of ERK2 in the caudal caudate putamen region of the striatum [34]. Clozapine has also been shown to activate levels of ERK1/2 in the prefrontal cortex region, by interaction with the epidermal growth factor (EGF) receptor [33]. This could have implications in regulating synaptic plasticity and connectivity, which are important physiological features dysregulated in schizophrenia [62,63].

In conclusion, this study has identified changes in the expression of key regulators involved in the activity of the dopamine D2 receptor in the striatum as a result of chronic treatment with the allosteric modulator, PAOPA. As hyperdopaminergia has been linked to certain symptoms of schizophrenia, this study brings to light the importance of understanding the effects of therapeutics on GPCR regulatory and downstream proteins. Changes in the expression of these regulatory molecules can have profound effects on receptor availability, and overall neurotransmission, thus making them vital therapeutic targets to attenuate pathological receptor phenotypes in various GPCR-related disorders. By identifying the affected signalling pathways, this study develops PAOPA into a prospective new antipsychotic drug which can lead to improved treatment of schizophrenia.
